# Assessment of the Characteristics and Associated Factors of Infectious Complications in Bullous Pemphigoid

**DOI:** 10.3389/fimmu.2020.01607

**Published:** 2020-07-23

**Authors:** Jia Chen, Xuming Mao, Wenling Zhao, Bingjie Zhang, Xinyi Chen, Chenyang Yu, Zehui Zheng, Hongzhong Jin, Li Li

**Affiliations:** ^1^Department of Dermatology, Peking Union Medical College Hospital, Chinese Academy of Medical Sciences and Peking Union Medical College, Beijing, China; ^2^Department of Dermatology, University of Pennsylvania, Philadelphia, PA, United States; ^3^Department of Dermatology, Shunyi Maternal and Children's Hospital of Beijing Children's Hospital, Beijing, China

**Keywords:** pemphigoid, infections, outpatients, corticosteroids, serum albumin

## Abstract

**Objectives:** The clinical outcome of bullous pemphigoid appears worse in patients with infectious complications, and assessment of the prevalence and risk factors of infectious complications could be necessary to plan preventative strategies and to instruct the treatment plans. We sought to determine the risk factors of infection and compare associated factors in inpatients and outpatients with different system infections.

**Design:** This is a single-centered retrospective study on the medical records of 252 patients from 2010 to 2018 at the dermatology department, Peking Union Medical College. Medical profiles of medical history, diagnosis, infectious complications, and treatment plans were analyzed. The associated factors were compared between the subgroups, including inpatients and outpatients, different body sites of infection.

**Results:** Of the total 252 patients with bullous pemphigoid (BP), 81 patients (81/252, 32.1%) had infectious complications. Forty-eight patients died from pulmonary infections (11/48, 22.9%), cardiovascular diseases (6/48, 12.5%), and other diseases. Infections were most frequently found in skin/mucosa (44/252, 17.5%), respiratory system (32/252, 12.7%), and blood (10/252, 4.0%). On multivariate analysis, risk factors of infections in BP were maximal control dose of corticosteroids (OR 2.539, 95% CI 1.456–4.430, *p* = 0.001), low serum albumin level (OR 2.557, 95% CI 1.283, 5.092, *p* = 0.007), hospitalization (OR 4.025, 95% CI 2.289, 7.079, *p* < 0.001), comorbidities including respiratory disease (OR 4.060, 95% CI, 1.861, 8.858, *p* < 0.001), eye disease (OR 4.431, 95% CI 1.864, 10.532, *p* < 0.001), and diabetes (OR 2.667, 95% CI 1.437, 4.949, *p* = 0.002). The rate of infection was significantly higher in inpatients compared to that in outpatients (54.0 vs. 20.6%, *p* < 0.001), with diverse risk factors. Mucocutaneous infections were associated with a maximal control dose of corticosteroid and other dermatoses. Respiratory infections were related to respiratory disease and old age, and hematologic infection was associated with low serum hemoglobin levels and mucosal involvement of BP. Both of them were associated with mucosal involvement of BP and high titer anti-BP180 antibody.

**Conclusions:** Infectious complications of bullous pemphigoid are common and are associated with mucosal involvement of BP, more comorbidities, the higher dose of corticosteroids, and the lower level of serum albumin.

## Introduction

Bullous pemphigoid (BP) is an autoimmune bullous skin disorder commonly identified in the elderly population. It is an autoantibody-induced cutaneous inflammatory disease against BP180 or BP230 at the dermal-epidermal junctions (1, 2). The annual incidence rate of BP has been increasing steadily in the elderly and general populations (3), and the BP patients were reported to have a significantly increased risk of death compared to the control subjects (4–7). Conditions such as older age, poor general condition, dementia, comorbidities, and high-dose corticosteroids have been reported to be the predisposing factors for death (8–11). Prior studies have indicated that infection is the leading cause of death in BP patients. In a published series, almost all BP patients treated with corticosteroids had at least one localized or systemic infection during the follow-up period, and 43% of those patients experienced systemic infections that require hospitalization or lead to death (12). Moreover, approximately one-third of BP patients developed localized skin infections, with 10% of fatal necrotizing fasciitis at 1 year after treatment with topical corticosteroids (13), supporting that infections occurring after the onset of BP tended to worsen the clinical outcomes (14–16). Consequently, the evaluation of the prevalence and risk factors of infectious complications could be indispensable for better preventative strategies and treatment plans with corticosteroids or other immunosuppressive drugs for these patients (14).

Given the importance of infections in BP prognosis and management, only a few studies have been published on the risk factors of infection (12, 17, 18). Those studies were limited with a small number of patients and emphasized the severe infections contributing to hospitalization or mortality. Moreover, previous researches focused mainly on inpatients but less on outpatients. In the current work, we enrolled a relatively large cohort of 252 BP patients, including 87 inpatients and 165 outpatients. The goal of our study is to: (1) retrospectively analyze and compare the clinical characteristics of infected BP patients in inpatients and outpatients, (2) determine the risk factors of infection by analyzing the comorbidities, blood test results and treatment choices, and (3) compare the associated factors in patients with infections at different body sites. The results from our BP patients' medical records were analyzed, discussed, and compared to those from previous studies.

## Materials and Methods

### Patients

Following the principles of the Declaration of Helsinki, this study was approved by the Ethical Committee of Peking Union Medical College Hospital (S-K965). We identified patients with bullous pemphigoid between 2010 and 2018 at the Department of Dermatology, PUMCH. The diagnosis of bullous pemphigoid (BP) was based on the S2k guideline for the diagnosis of bullous pemphigoid (19). Patients with one of the three constellations below were included in our study: (1) compatible clinical picture, and either corresponding histopathology or positive direct IF microscopy, and either epidermal binding of IgG in indirect IF microscopy (on split skin or monkey esophagus) or reactivity with BP180 antibody; (2) clinical picture with tense blisters, and epidermal binding of IgG in indirect IF microscopy, and either corresponding histopathology or reactivity with BP180 antibody; (3) clinical picture with tense blisters, and reactivity with BP180 antibody higher than 27 U/ml. Patients without a record of medical history, basic serologic tests, and treatment plans for further analysis were ruled out. Results of the BP230 antibody were obtained only in one patient, so it was not considered as a part of diagnostic criteria. We included and followed up both outpatients and inpatients with BP until Dec 2019.

### Data Collection

Patients' files were reviewed to collect baseline information (i.e., gender, age, and birthplace), demographic characteristics and skin lesion distribution, results of clinical tests on the diagnosis of diseases before infectious complications, the history of past illness, comorbidities, and the treatment plans. Infections that did not result in clinical symptoms were not included because of the low number and thus were less likely to affect clinical prognosis in patients with BP. The information for disease severity was not available for analysis, but the skin lesions were documented in detail in the medical records.

### Statistical Analysis

Data were investigated first for all inpatients and outpatients diagnosed with BP and were further analyzed for all patients with infectious complications. Comparisons of the associated factors between subgroups (different sites of infection) were conducted. Descriptive statistics were applied to report the baseline characteristics, demographics, test results, past medical history, comorbidities, and treatment variables (genres of immunosuppressant, maximum control dose of corticosteroid). Student's *t*-test or Mann-Whitney U test was used in continuous outcomes with a normal distribution or not. Categorical data were first analyzed with the Chi-square test or Fisher's exact test and then reported as the relative risk with 95% confidence intervals and *P* values. We made a multivariate analysis, using binary outcome and incorporating the factors found significant by univariate analysis and those deemed clinically significant. Statistical analyses were performed using software (SPSS, Version 25, IBM Corp., Armonk, NY; RStudio, Version 1.2.1335). All tests were two-tailed, and *p* < 0.05 was considered statistically significant.

## Results

### Baseline Characteristics

We searched for the hospital information system and found that 383 patients were initially diagnosed with BP from 2010 to 2018. One hundred two of them were ruled out because of doubtful diagnosis, and 29 of them were excluded due to a lack of data for further analysis. Eventually, a total number of 252 patients were included, and 81 of them were diagnosed with infectious complications after BP onset. Among them, 48 patients died due to pulmonary infections (11/48, 22.9%), cardiovascular diseases (6/48, 12.5%), cerebral infarction (5/48, 10.4%), BP relapse (4/48, 8.3%), cancer (3/48, 6.3%), digestive diseases (2/48, 4.2%), and other unknown reasons (17/48, 35.4%). The patients were followed up for an average of 2.9 ± 0.2 years from the beginning of diagnosis.

The female to male ratio was 1.2:1, with an average age of 67.2 years old at BP onset. The median interval from the onset of BP to diagnosis was 9.1 months (Table 1). 67.1% of the patients only had skin involvement, and 24.2% of the patients had both skin and mucosal involvement of BP. The oral mucosa was the most frequently affected mucosa in BP. All patients were followed up in our outpatient clinic. 34.5% were admitted as inpatients for an average of 23.2 days in the hospital. 74.6% of the patients were treated with oral or intravenous corticosteroids, 52.0% with immunosuppressants, and 3.6% with IVIG. One hundred fifteen patients were treated with only one, and 16 patients with two or three immunosuppressants (Supplementary Table 1). Additional therapy adjuvants include minocycline, nicotinamide, and topical corticosteroids.

**Table 1:** Demographic and clinical features of all BP patients.

**Features**	***N* = 252**
Age/year (average, median)	67.2 ± 1.0, 69.1
Gender	
Male (*n*, %)	137 (54.4)
Female (*n*, %)	115 (45.6)
Duration before diagnosis of BP/month	9.1 ± 1.2, 3.0
Follow-up period/year	2.9 ± 0.2, 2.5
Distribution	
Skin only (*n*, %)	169 (67.1)
Skin and mucosa (*n*, %)	61 (24.2)
Mucosa only (*n*, %)	5 (2.0)
Hospitalization	
Number of inpatients (*n*, %)	87 (34.5)
Duration/day	23.2 ± 1.9, 19.5
Treatments	
Corticosteroids (*n*, %)	188 (74.6)
Immunosuppressants (*n*, %)	131 (52.0)
IVIG	9 (3.6)
Infections (*n*, %)	81 (32.1)

*In total, 252 patients were included. The average ± SD and median values were calculated for age, duration before BP diagnosis, follow-up period, and hospitalization duration, respectively. For each of the other categories, the number (n) and percentage (%) of patients were listed. IVIG, intravenous immunoglobulin*.

### Infectious Complications

Infectious complications occurred in 81 patients (81/252, 32.1%), of which 50 patients (50/81, 61.7%) had infections within the first year after BP diagnosis. The median duration between BP diagnosis and infection onset was 5 months.

Localized infections are infections in the skin, oral mucosa, and vulva, and systemic infections are infections in the lung (including TB infection, TB reactivation, and pneumonia), upper respiratory tract, urinary tract (UTI), digestive system, blood infection, and central nervous system infection (CNSI) (Table 2). Diagnosis of infectious complications was based on clinical manifestations and lab tests such as microbial cultivation, antimicrobial susceptibility tests, DNA, and specific IgG detection. Cutaneous and respiratory infections were the most commonly observed infections. The most common pathogens identified in these patients were the staphylococcus, candida, and cytomegalovirus (CMV), respectively. CNSI was diagnosed in one patient based on clinical manifestations, the biochemical tests of her spinal fluid, and the blood cultures with Listeria monocytogenes.

**Table 2:** Sites of infectious complications and pathogens.

**Body sites**	**Total (*N* = 81)**	**Specimen**	**Species (numbers of cases)**
**Skin and perineum**	**40**	**Pus**	*Staphylococcus aureus* (4), *Pseudomonas aeruginosa* (3), *Corynebacterium* (2), *Staphylococcus haemolyticus* (2), *Staphylococcus epidermidis* (2), *Enterobacter cloacae* (2), *Enterococcus faecium* (1), *Enterococcus raffinosus* (1), *Staphylococcus intermedius* (1), *Streptococcus pyogenes* (1), β-hemolytic streptococcus (1), *Acinetobacter* (1), *Serratia marcescens* (1), *Proteus mirabilis* (1), *Prevotella melaninogenica* (1), *Actinomyces odontolyticus* (1), *Candida albicans* (1), *Candida parapsilosis* (1), *Candida glabrata* (1), CMV (1)
**Mouth**	**4**	**Mouth swab**	*Candida albicans* (2)
		**Throat swab**	*Staphylococcus aureus* (1), *Candida albicans* (1), *Mycoplasma chlamydia* (1)
**Respiratory system**	**32**	**Sputum**	*Candida albicans* (2), *Acinetobacter baumannii* (1), *Corynebacterium* (1), *Staphylococcus aureus* (1), *Streptococcus pneumonia* (1), *Moraxella catarrhalis* (1)
Pneumonia	23		
Upper respiratory infection	5		
Pulmonary tuberculosis	4		
**Urinary system**	**8**	**Urine**	*Escherichia coli* (2), *Candida albicans* (2), *Candida tropicalis* (2), *Klebsiella pneumonia* (1), *Enterobacter gergoviae* (1), *Enterococcus faecalis* (1), *Proteus mirabilis* (1)
**Digestive system**	**4**	**Feces**	*Candida tropicalis* (1)
Hepatitis B	2		
Dysentery	1		
Diarrhea	1		
**Blood**	**10**	**Blood**	CMV (3), *Staphylococcus aureus* (2), *Enterococcus faecalis* (2), *Enterobacter cloacae* (1), *Staphylococcus haemolyticus* (1), *Listeria monocytogenes* (1), *Serratia marcescens* (1), *Pseudomonas aeruginosa* (1), EBV (1)
Bacteremia	9		
Septic shock	1		
**Central nervous system**	**1**		

*81 of 252 patients with BP had infectious complications. The left column of the table shows the types of infection that occurred in different organ systems, including skin or perineum, mouse, respiratory system, urinary system, digestive system, blood, and central nervous system. The right column revealed the tested specimens and identified pathogens from the body sites of these patients. A patient may have infections at different body sites*.

### Risk Factors for Infectious Complications

**Figure 1:**
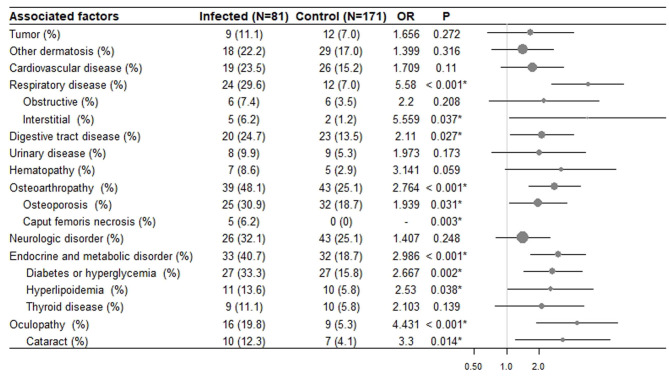
The risk of infection in BP patients with different comorbidities. In all 252 BP patients, 171 had no infectious complication, and 81 had infections. The odds ratios (OR) and *p* values were calculated. *Denotes statistical significance (*p* < 0.05). Forest plots show odds ratios of different comorbidities with a 95% confidence interval.

On univariate analysis, significant risk factors for developing infectious complications include respiratory disease (*p* < 0.001), digestive disease (*p* = 0.027), osteoarthropathy (*p* < 0.001), endocrine and metabolic disease (*p* < 0.001) oculopathy (*p* < 0.001) (Figure 1). Laboratory biochemical tests showed that the serum albumin level was lower in the infected group (*p* = 0.004) (Supplementary Figure 1). No significant difference was found in other serum lab tests, tumors, neurologic disorders, urinary diseases, hematological diseases, other dermatoses, and cardiovascular diseases.

Additionally, patients with mucosal involvement of BP (OR 2.443, 95% CI 1.356, 4.440; *p* = 0.003) and hospitalization (OR 4.025, 95% CI 2.289, 7.079; *p* < 0.001) were more likely to have infectious complications. The maximal control doses of oral corticosteroids were higher in the infected group (OR 2.539, 95% CI 1.456, 4.430; *p* = 0.001). Infectious diseases were not related to applying the immunosuppressants or not (*p* = 0.062). The duration of hospitalization in inpatients was also longer in the infected group (27.8 d vs. 18.1 d, *p* = 0.010). The gender distribution (*p* = 0.422), average age (*p* = 0.385) of patients in two groups were similar.

On multivariate analysis, the maximal control dose of corticosteroids (OR 2.539, 95% CI 1.456–4.430, *p* = 0.001), low serum albumin level (OR 2.557, 95% CI 1.283, 5.092, *p* = 0.007), hospitalization (OR 4.025, 95% CI 2.289, 7.079, *p* < 0.001), comorbidities including respiratory disease (OR 4.060, 95% CI, 1.861, 8.858, *p* < 0.001), eye disease (OR 4.431, 95% CI 1.864, 10.532, *p* < 0.001), and diabetes (OR 2.667, 95% CI 1.437, 4.949, *p* = 0.002) remained significant. Osteoarthropathy often occurred after the corticosteroid treatment, so it was not regarded as an independent risk factor for developing infectious complications.

### The Difference of Infections in Inpatients and Outpatients

Eighty-seven inpatients and 165 outpatients were followed up. Among 81 patients with infectious diseases, 47 (54.0% of the inpatients) of them were inpatients, and 34 (20.6% of the outpatients) outpatients. There was a significantly higher rate of infections in inpatients compared to that of outpatients (*p* < 0.001).

We further analyzed the basic characteristics, all comorbidities, and results of blood tests of the two groups listed previously in Table 1, Figure 1, and Supplementary Figure 1. The comparison was made between the infected group and the control group in inpatients and outpatients, respectively. The characteristics of infected group of inpatients and outpatients were also compared (Table 3).

**Table 3:** Infectious complications in BP inpatients and outpatients.

**Group of patients**	**Inpatients (*****N*** **=** **87)**			**Outpatients (*****N*** **=** **165)**			***P***
	**Infected**	**Control**	***p***	**Infected**	**Control**	***p***	
Number (%)	47 (54.0)	40 (46.0)	–	34 (20.6)	131 (79.4)	–	<0.001^*^
Gender (M/F)	31/16	21/19	0.273	16/18	69/60	0.565	0.112
Age of BP onset/y	66.9 ± 1.9 67.1	59.3 ± 2.6, 60.2	0.020^*^	70.6 ± 3.2, 76.9	68.9 ± 1.3, 70.9	0.249	0.098
Duration before diagnosis/m	10.7 ± 3.0, 4.0	6.1 ± 1.3, 3.0	0.928	7.8 ± 4.0, 3.0	9.9 ± 1.8, 3.0	0.430	0.469
Duration before infection/m	14.4 ± 3.2, 5.2	–	–	15.5 ± 3.3, 5.2	–	–	0.105
Duration of Hospitalization/d	27.8 ± 3.1, 26.0	18.1 ± 1.7, 17.0	0.010^*^	–	–	–	–
Mucosal involvement of BP	21 (44.7%)	14 (35.0%)	0.381	11 (32.4%)	20 (15.3%)	0.044^*^	0.271
Control dose of corticosteroid (mg/d)	53.3 ± 6.4, 40.0	42.4 ± 4.3, 40.0	0.534	26.8 ± 3.1, 30.0	24.3 ± 2.4, 20.0	0.213	<0.001^*^
Auxiliary exam							
Anti-BP180	75.1 ± 8.6, 74.0	47.7 ± 8.7, 33.0	0.015^*^	64.9 ± 12.0, 23.0	57.7 ± 5.2, 43.0	0.699	0.300
Serum Hb	131.8 ± 2.9, 138.0	130.5 ± 3.2, 132.0	0.563	128.9 ± 3.3, 134.0	136.8 ± 1.8, 135.0	0.033^*^	0.372
Serum Alb	34.9 ± 1.1, 36.0	37.4 ± 0.8, 37.0	0.071	41.0 ± 1.8, 39.0	40.9 ± 0.7, 41.0	0.215	0.014^*^
Comorbidities							
Diabetes	17 (36.2%)	10 (25.0%)	0.262	10 (29.4%)	17 (13.0%)	0.021^*^	0.524
Hyperlipidemia	11 (23.4%)	2 (5.0%)	0.018^*^	0 (0.0%)	8 (6.1%)	0.208	0.002^*^
Gastritis	1 (2.1%)	2 (5.0%)	0.592	4 (11.8%)	2 (1.5%)	0.017^*^	0.156
Osteoarthropathy	22 (46.8%)	14 (35.0%)	0.265	17 (50.0%)	29 (22.1%)	0.001^*^	0.777
Respiratory disease	15 (31.9%)	2 (5.0%)	0.002^*^	4 (11.8%)	10 (7.6%)	0.490	0.035^*^
Neurologic disorder	12 (25.5%)	14 (35.0%)	0.336	14 (11.8%)	29 (22.1%)	0.024^*^	0.137

For inpatients and outpatients, the difference between patients with infections (infected) and without infection (control) was compared, respectively. The p values were calculated in columns 4 and 8, respectively. The difference between the infected group of inpatients and outpatients was also estimated with p values (last column) (

* *denotes statistical significance or p < 0.05). BP, bullous pemphigoid*.

In the inpatient group, old age, high anti-BP180 titer, hyperlipidemia, and respiratory disease were associated with a higher incidence of infectious complications. In the outpatient group, low serum hemoglobin levels, mucosal involvement of BP, comorbidities of diabetes, gastritis, osteoarthropathy, and neurologic disorder were associated with a higher incidence of infectious complications. Compared to the outpatients with infections, the inpatients with infections were treated with a significantly higher control dose of corticosteroids (*p* < 0.001) and had a higher incidence of hyperlipidemia (*p* = 0.002) and respiratory disorders (*p* = 0.035). There is no significant difference in gender and onset-time variation of BP or infection.

### The Difference Between Infections in Different Organ Systems

Infections in the respiratory system, hematological system, skin, and mucosa were analyzed, but not in the digestive system and urinary system because of the small sample size (<10). Patients with infection at each site were compared with patients in the control group (uninfected BP patients at the same site).

Respiratory infections were related to advanced age (*p* = 0.008) (Table 4). The dose of corticosteroids was associated with an increased tendency of infection, and subgroups analysis showed that the control dose of corticosteroids was significantly correlated with mucocutaneous infections (*p* = 0.004) but not with respiratory (*p* = 0.268) or bacteremia (0.062) (Table 4). Mucosal involvement of BP was associated with only hematologic infections (*p* < 0.001) but not the other two infections (*p* = 0.068). The incidence of hospitalization was significantly higher in patients with mucocutaneous infections (*p* < 0.001) or hematologic infections (*p* < 0.001), but not in patients with respiratory infections (*p* = 0.067).

**Table 4:** Infectious complications in different organ systems, including mucocutaneous, respiratory, and blood.

	**Mucocutaneous**			**Respiratory**			**Blood**		
	**Infected**	**Control**	***p***	**Infected**	**Control**	***p***	**Infected**	**Control**	***p***
	**(*N* = 44)**		**(*N* = 208)**	**(*N* = 32)**		**(*N* = 220)**	**(*N* = 10)**	**(*N* = 242)**	
Gender (M/F)	1.59	1.05	0.351	1.46	1.19	0.596	0.30	1.28	0.118
Age of BP onset/year	64.6 ± 2.2, 65.1	67.9 ± 1.1, 70.5	0.140	72.1 ± 3.5, 76.9	66.2 ± 1.1, 67.2	0.008^*^	63.6 ± 2.5, 64.4	67.5 ± 1.0, 70.4	0.202
Duration before diagnosis/months	9.4 ± 3.7, 3.0	9.1 ± 1.3, 3.0	0.224	10.4 ± 3.6, 5.0	8.9 ± 1.3, 3.0	0.707	3.3 ± 0.9, 3.0	9.4 ± 1.3, 3.0	0.398
Duration before infection/months	16.3 ± 3.4, 3.7	-	-	15.0 ± 3.2, 5.1	-	-	2.1 ± 0.3, 2.1	-	-
Control dose of corticosteroids (mg/day)	49.2 ± 7.1, 40.0	29.8 ± 1.9, 30.0	0.004^*^	34.8 ± 3.9, 33.8	33.1 ± 2.3, 30.0	0.268	69.8 ± 25.0, 40.0	31.8 ± 1.9, 30.0	0.062
Mucosal involvement of BP (%)	17 (38.6)	49 (23.6)	0.068	13 (40.6)	53 (24.1)	0.068	9 (90.0)	57 (23.6)	<0.001^*^
Auxiliary exam									
Hb <130 g/L (%)	27 (61.4)	106 (51.0)	0.960	17 (53.1)	116 (52.7)	0.269	3 (30.0)	130 (53.7)	0.034^*^
Anti-BP180 > 50 U/L (%)	18 (40.9)	62 (29.8)	0.747	16 (50.0)	64 (29.1)	0.023^*^	8 (80.0)	72 (29.8)	0.002^*^
Comorbidities									
Other dermatosis (%)	13 (29.5)	34 (16.3)	0.043^*^	7 (21.9)	40 (18.2)	0.625	2 (20.0)	45 (18.6)	1.000
Respiratory diseases (%)	10 (22.7)	21 (10.1)	0.021^*^	9 (28.1)	22 (10.0)	0.008^*^	5 (50.0)	26 (10.7)	0.003^*^
Anemia (%)	5 (11.4)	5 (2.4)	0.017^*^	4 (12.5)	6 (3.0)	0.026^*^	2 (20.0)	8 (3.3)	0.054
Hospitalization									
Number (%)	28 (63.6)	54 (26.1)	<0.001^*^	15 (46.9)	67 (30.5)	0.067	9 (81.2)	73 (30.2)	<0.001^*^
Duration/day	27.9 ± 3.7, 25.5	20.7 ± 2.0, 17.0	0.045^*^	26.9 ± 5.7, 21.0	22.4 ± 1.9, 19.0	0.556	26.8 ± 5.6, 28.0	22.7 ± 2.0, 18.0	0.350

*In each type of infection, the difference between patients with infection (infected) and without infection (control) was calculated as p-values, respectively*.

* *Denotes statistical significance or p <0.05*.

Analysis of all comorbidities and results of blood tests in subgroups confirmed that most of the associated factors were the same as previously analyzed. We listed three comorbidities, serum hemoglobin level, and BP180 antibody titer in Table 4, to reveal the relationship between the infection and the abnormality at the same site. We found mucocutaneous infections were associated with other dermatoses (*p* = 0.043); respiratory infections were related to respiratory disease (*p* = 0.008), and hematologic infections were associated with low serum hemoglobin level (*p* = 0.034).

## Discussion

In our study, we have shown that 32.1% of patients with bullous pemphigoid were affected by infectious complications. The median duration before infection was 5 months, with 61.7% occurring in the first year after BP diagnosis. Mucocutaneous (17.5%) and respiratory infections (12.7%) were the most frequent, followed by bacteremia (4.0%). The most common pathogens were *S. aureus* and *C. albicans*. Factors associated with developing infections on univariate analysis were the comorbidities of multiple systems, mucosal involvement of BP, more extended periods of hospitalization, higher maximal control doses of corticosteroid, and a lower level of serum albumin. On multivariate analysis, a higher dose of corticosteroid, a lower level of serum albumin, the experience of hospitalization, and comorbidities, including respiratory disease, eye disease, and diabetes, remained significant.

The inpatients were more vulnerable to infectious complications than outpatients (54 vs. 20.6%, p <0.001). A plausible explanation could be that the inpatients had a higher maximal control dose of corticosteroid and a higher frequency of respiratory diseases—both are significant risk factors of infections in BP. On subgroup analysis, mucocutaneous infections were associated with corticosteroids and other dermatoses. As predicted, respiratory infections were related to respiratory disease. Hematologic infections were associated with low serum hemoglobin levels, although the significance and mechanism remain to be investigated in the future.

Interestingly, mucosal involvement of BP was significantly correlated with hematologic infections or sepsis, which could contribute to higher mortality of BP patients. Moreover, we found that the ratio of patients with mucosal involvement of BP was significantly higher in the infected outpatients than in the uninfected outpatients with BP. Kridin and Bergman have shown that a majority of mucosal involvements of BP occur in the mouth and are related to extensive cutaneous disease, lower peripheral eosinophilia, and more aggressive treatment (20). In our study, the association between infectious complications and increased doses of corticosteroids was also significant, suggesting that the correlation between mucosal involvement of BP and infection is dependent on treatment.

As the BP patients with cardiovascular diseases, dementia, and stroke proved to be at a higher risk of mortality (21, 22), we further analyzed the correlation between comorbidities and infectious complications. Our results indicate that infections are significantly higher in BP patients with comorbidities such as diabetes and respiratory diseases. A nationwide study from the USA found that a higher number of chronic conditions (RA, SLE, or type I diabetes) was a risk factor of severe infections in BP patients (18). A retrospective study from Singapore found that functional impairment (Karnofsky score <60, CCIS ≥6) and dementia are risk factors of infection in a cohort of 97 BP patients (17). The severity of disease and diabetes have also been reported to be directly related to more infections in bullous diseases (23, 24). Nevertheless, we cannot exclude the possibility that patients with infectious complications tend to receive more medical examinations with more detailed documentation of medical history, which may contribute to the higher number of comorbidities in BP patients.

Notably, we found that lower serum Alb level and higher BP180 antibody titer were significantly associated with the development of infectious complications in BP patients. A higher BP antibody level could cause a more severe disruption of the skin barrier function, which could lead to more infections. Our results indicated that a higher titer BP180 antibody was associated with respiratory and hematologic infections. The serum alb level is a marker of the nutrition level. Research published recently pointed out that patients at risk of malnutrition were more likely to have a healthcare-associated infection, with malnutrition assessed by BMI, recent weight loss, and dietary intake (25). A case report and retrospective analysis from Japan discovered that higher BP180 antibody titer resistant to corticosteroids treatment was a risk factor for developing CMV infection in BP patients (26). The infection by Varicella Zoster virus (VSV) that also belongs to the family of herpes virus as CMV was reported to enhance BP180 antibody production (27). Additionally, reduction in serum WBC, PLT, and increase in ALT have also been reported to be risk factors (26).

Some therapies, particularly corticosteroids, could lead to more infectious complications in BP patients. A retrospective study in Mayo Clinic revealed that all patients with autoimmune bullous diseases and taking systemic corticosteroids had an infection during the follow-up (12). The risk of using topical corticosteroids should not be neglected, especially in patients with diabetes and mucocutaneous infection (13). Case reports and literature reviews have demonstrated that corticosteroids and ibrutinib may be associated with opportunistic fungal and virus infections (28–31). The aforementioned nationwide study in the USA also pointed out that severe infections in inpatients with pemphigus and pemphigoid were associated with prolonged hospitalization and increased mortality (18). In our preliminary clinical observations, the choice of hospitalization and the duration in the hospital were not risk factors for infection in bullous pemphigoid, potentially because infectious complications can prolong hospital stay in the first place.

Other risk factors for infections in BP patients include female, non-white race, and poor economic condition, as described in the previous studies (18). The circulating anti-centrosome antibody has also been reported associated with infection (32). Interestingly, two previous retrospective analyses have shown that the incidence of pneumocystis pneumonia (PCP) infection was lower than expected in patients with the autoimmune bullous disease (33, 34).

## Conclusion and Limitations

BP patients have a high risk of infectious complications, which are associated with mucosal involvement of BP, more comorbidities, a higher dose of corticosteroid, and a lower level of serum albumin. The inpatients have a higher risk of infection than the outpatients. The main limitation of this study originated from the retrospective nature of data collection. The factors that may affect the interpretation of results are the detailedness of documentation of hematological findings, the accuracy of recording of infected sites, and comprehensiveness of screenings for pathogens. Another limitation could be that most of our patients were residents from the north of China, although they were from different provinces. However, our study comprised a relatively large cohort of both inpatients and outpatients and described the pathogens of infectious complications. Infectious complications were analyzed further in subgroups as mucocutaneous, respiratory, and hematological infections. To our knowledge, this is the first study for characterizing infectious complications in BP outpatients. Our results should provide insight into better management of BP patients during the long-term, chronic disease course. In the future, a multicentric study with a larger sample size will be needed to verify our study.

## Data Availability Statement

All datasets presented in this study are included in the article/Supplementary Material.

## Ethics Statement

The studies involving human participants were reviewed and approved by the Ethical Committee of Peking Union Medical College Hospital (S-K965). Written informed consent for participation was not required for this study in accordance with the national legislation and the institutional requirements.

## Author Contributions

JC performed the data analysis and drafted the manuscript. XM and WZ revised and edited the manuscript. BZ, XC, CY, and ZZ collected information and were responsible for data curation. HJ provided resources and information. LL conceived the idea, revised, and edited the manuscript. All authors read and made final approval of the manuscript. All authors contributed to the article and approved the submitted version.

## Conflict of Interest

The authors declare that the research was conducted in the absence of any commercial or financial relationships that could be construed as a potential conflict of interest.

## Supplementary Material

Supplementary Figure 1 Results of serum laboratory tests. The values of the variables, as indicated on the bottom of each figure, were plotted. The difference of uninfected patients (control) and infected patients were compared, as indicated with the P-value on the top of each figure. WBC, white blood cell; Ly#, cell count of lymphocyte; Ly%, percentage of lymphocyte; Eos#, cell count of eosinophilia; Eos%, percentage of eosinophilia; Alb, serum albumin; PAB, serum pre-albumin; Hb, hemoglobin.

Supplementary Table 1 Application of immunosuppressant in all BP patients. The numbers (*n*) and percentages(%) of patients treated with each of the immunosuppressants were calculated(left column). The Right column shows the numbers(*n*) and percentages(%) of patients treated with one, two, or three types of immunosuppressants, respectively. MTX, methotrexate; MMF, Mycophenolate mofetil; CTX, cyclophosphamide; CsA, cyclosporine.
