# Supplementary figures and images for: Assessment of the Characteristics and Associated Factors of Infectious Complications in Bullous Pemphigoid
Source: Front Immunol. 2020 Jul 23;11:1607. doi: 10.3389/fimmu.2020.01607 (PMC7390841; doi:10.3389/fimmu.2020.01607)

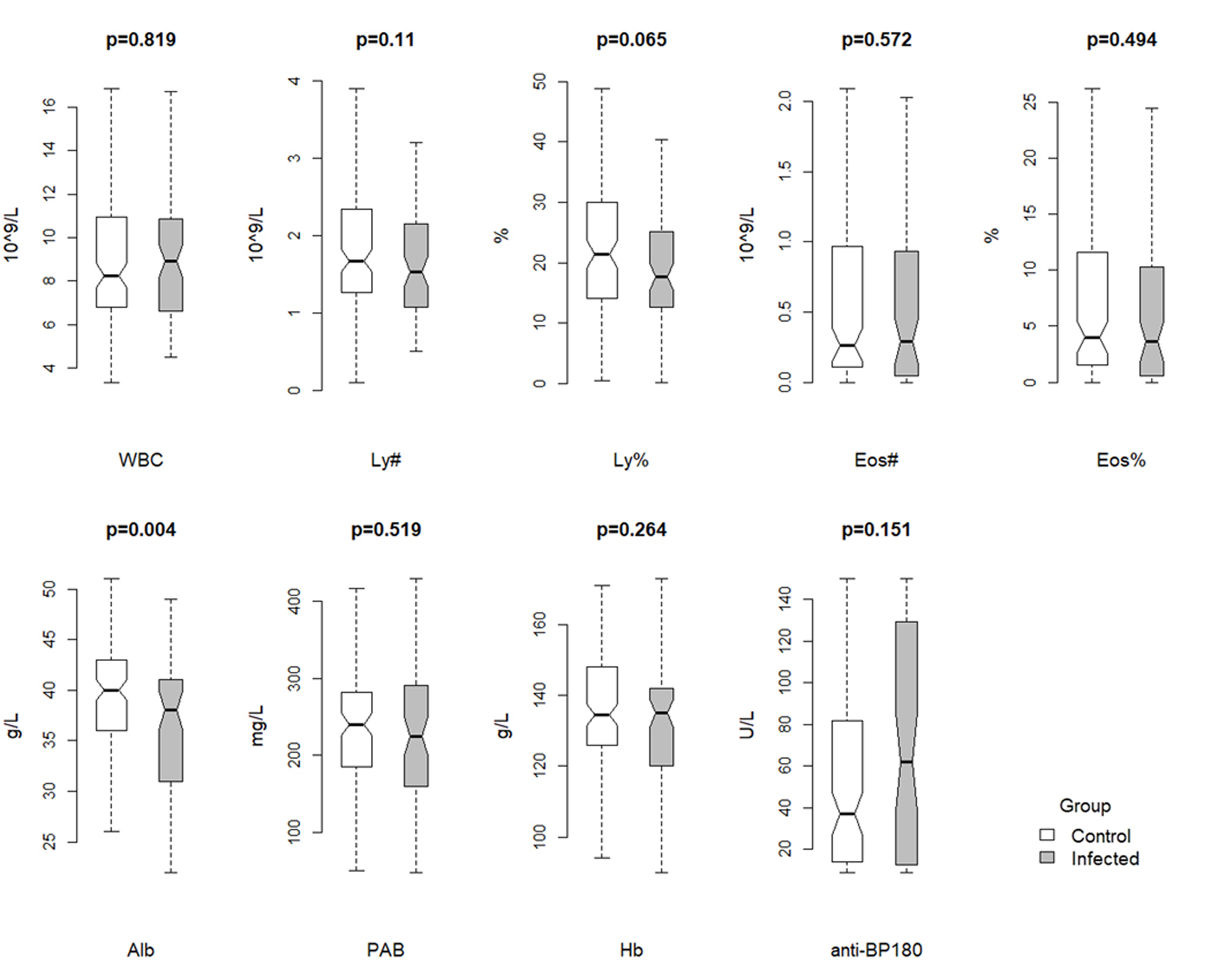

Supplement: Supplementary Figure 1 — Results of serum laboratory tests. The values of the variables, as indicated on the bottom of each figure, were plotted. The difference of uninfected patients (control) and infected patients were compared, as indicated with the P-value on the top of each figure. WBC, white blood cell; Ly#, cell count of lymphocyte; Ly%, percentage of lymphocyte; Eos#, cell count of eosinophilia; Eos%, percentage of eosinophilia; Alb, serum albumin; PAB, serum pre-albumin; Hb, hemoglobin. [file Image_1.jpeg]
